# Determination of Urinary Neopterin/Creatinine Ratio to Distinguish Active Tuberculosis from Latent* Mycobacterium tuberculosis* Infection

**DOI:** 10.1155/2016/5643853

**Published:** 2016-06-28

**Authors:** Michael Eisenhut, Dougal S. Hargreaves, Anne Scott, David Housley, Andrew Walters, Rohinton Mulla

**Affiliations:** ^1^Luton & Dunstable University Hospital NHS Foundation Trust, London LU4 ODZ, UK; ^2^Institute of Child Health, University College London, London WC1N 1EH, UK

## Abstract

*Background*. Biomarkers to distinguish latent from active* Mycobacterium (M.) tuberculosis* infection in clinical practice are lacking. The urinary neopterin/creatinine ratio can quantify the systemic interferon-gamma effect in patients with* M. tuberculosis* infection.* Methods*. In a prospective observational study, urinary neopterin levels were measured by enzyme linked immunosorbent assay in patients with active tuberculosis, in people with latent* M. tuberculosis* infection, and in healthy controls and the urinary neopterin/creatinine ratio was calculated.* Results*. We included a total of 44 patients with* M. tuberculosis* infection and nine controls. 12 patients had active tuberculosis (8 of them culture-confirmed). The median age was 15 years (range 4.5 to 49). Median urinary neopterin/creatinine ratio in patients with active tuberculosis was 374.1 micromol/mol (129.0 to 1072.3), in patients with latent* M. tuberculosis* infection it was 142.1 (28.0 to 384.1), and in controls it was 146.0 (40.3 to 200.0), with significantly higher levels in patients with active tuberculosis (*p* < 0.01). The receiver operating characteristics curve had an area under the curve of 0.84 (95% CI 0.70 to 0.97) (*p* < 0.01).* Conclusions*. Urinary neopterin/creatinine ratios are significantly higher in patients with active tuberculosis compared to patients with latent infection and may be a significant predictor of active tuberculosis in patients with* M. tuberculosis* infection.

## 1. Introduction

A biomarker to discriminate active from latent* Mycobacterium tuberculosis* infection could help in exclusion of occult pulmonary and extrapulmonary tuberculosis. It could avoid undertreatment of patients treated as latent tuberculosis on grounds of a normal chest X-ray which may leave both active pulmonary and extrapulmonary tuberculosis undetected. There is no laboratory test available in clinical routine which can distinguish latent from active* Mycobacterium (M.) tuberculosis* infection. Scientific research so far has identified the cytokine profile produced by T-lymphocytes as indicator of active versus latent* M. tuberculosis* infection [1, 2]. Proteomic fingerprinting of serum has led to identification of proteins present in the serum of patients with active tuberculosis [3]. Application of T-cell phenotyping or proteomic fingerprinting of serum to diagnose active tuberculosis is currently limited to research settings and may be difficult to translate into an affordable test usable in clinical routine particularly in developing countries.

Neopterin in chemical nomenclature 2-amino-4-hydroxy-6-(D-erythro-1′,2′,3′-trihydroxypropyl)-pteridine is derived from guanosine triphosphate via the activity of guanosine triphosphate cyclohydrolase I and is produced by activated monocytes, macrophages, dendritic cells, and endothelial cells and to a lesser extent by renal epithelial cells, fibroblasts, and vascular smooth muscle cells upon stimulation mainly by interferon-gamma and to a lesser extent by interferon alpha and beta with its release being enhanced by tumor necrosis factor [4]. Neopterin is, after production, secreted unaltered in urine where it is concentrated. By calculating urinary neopterin/creatinine ratio, urinary levels are corrected for variations in urine production resulting for example from dehydration in a patient who is unwell. Urinary neopterin levels can be determined by a simple ELISA system [5] and a dipstick system has been developed to determine neopterin levels semiquantitatively in serum [6]. A previous study reported urinary neopterin/creatinine ratios in patients with active tuberculosis and found them to be higher than in patients with pneumonia and lung cancer [7]. Patients with moderately advanced disease had higher levels than patients with minimal disease.

Neopterin has previously been identified as a candidate biomarker in tuberculosis, which may be suitable as predictor of treatment effect and relapse [8]. Neopterin levels have not previously been investigated as a marker distinguishing active from latent* M. tuberculosis* infection.

The objectives of this study were to test the hypotheses that urinary neopterin/creatinine ratio is significantly higher in patients with active tuberculosis compared to people with latent* M. tuberculosis* infection and that urinary neopterin/creatinine ratios decrease significantly during treatment of active tuberculosis.

## 2. Methods

### 2.1. Study Population

Children and adults diagnosed with* M. tuberculosis* infection were recruited prospectively. Active tuberculosis was diagnosed following the definition by the guidelines of the National Institute of Health and Care Excellence of the United Kingdom [9] as patients with positive tuberculin skin test or positive interferon-gamma release assay and clinical, radiological, or culture evidence of tuberculosis. Latent* M. tuberculosis* infection was defined following the same guidance as positive tuberculin skin test and positive interferon-gamma release assay or only a positive interferon-gamma release assay with no clinical, radiological, or culture evidence of tuberculosis. Patients with latent* M. tuberculosis* infection were recruited after diagnosis and referral as part of contact investigations around patients with active pulmonary tuberculosis.

Healthy relatives testing negative by interferon-gamma release assay and/or tuberculin skin test during contact screening were recruited as controls. Excluded were patients with autoimmune disease, on immunosuppressant medication, or with current infections other than with* M. tuberculosis* or previously completed treatment for* M. tuberculosis* infection. The study was performed with ethical approval by the National Research Ethics Service of the United Kingdom and local Research and Development Department approval. Informed written consent was obtained from all patients and/or legal guardians of children included in the study.

### 2.2. Sample Collection and Laboratory Analysis

Urine neopterin/creatinine levels were measured before start of treatment. Changes of urinary neopterin/creatinine ratios were investigated during treatment in subgroups of patient with active and latent* M. tuberculosis* infection by repeating the measurement during clinic follow-up after commencement of treatment. Urinary creatinine levels were determined by the department of clinical biochemistry of the Luton & Dunstable University Hospital NHS Foundation Trust.

Urinary neopterin levels were determined by Enzyme Linked Immunosorbent Assay (ELISA) (IBL Gesellschaft fuer Immunchemie und Immunbiologie mbH, Hamburg, Germany) as described previously [5]. Employed was a solid phase ELISA based on the basic principle of a competitive ELISA. An unknown amount of antigen in the sample and a fixed amount of enzyme labelled antigen compete for the antibody-binding sites (rabbit-anti-neopterin). Both antigen-antibody complexes bind to the wells of the microtiter strips coated with a goat-anti-rabbit antibody. Unbound antigen is removed by washing. The intensity of the colour developed after the substrate incubation is inversely proportional to the amount of antigen in the sample. Laboratory analyses were determined by staff blinded to the status of study participants. IBL Gesellschaft fuer Immunchemie und Immunbiologie mbH had no influence on study planning or data analysis or interpretation.

### 2.3. Statistical Analysis

Based on a previous study [7], a sample size calculation was performed. For patients with active tuberculosis, a urine neopterin/creatinine ratio of 912.6 micromol/mol was taken and for patients with latent* M. tuberculosis* infection a level of 326.9 micromol/mol was taken, which was the median between patients with minimal disease and controls in this study and as standard deviation 451.4 micromol/mol from the group with minimal disease. To detect a difference between patients with active tuberculosis and people with latent* M. tuberculosis* infection with 90% power at a significance level of 5% based on these figures, it was determined that at least 9 patients with active tuberculosis and 18 people with latent* M. tuberculosis* infection are required.

Because of features of a nonparametric distribution, neopterin/creatinine ratios were compared using the Kruskal-Wallis test for multiple comparisons and the Mann-Whitney *U* test or Wilcoxon signed ranks test (paired samples) for comparison between two groups. To enable future sample size calculations, mean and standard deviation for urinary neopterin/creatinine ratios for patients with active and latent* M. tuberculosis* infection were also presented (see Appendix of Supplementary Material available online at http://dx.doi.org/10.1155/2016/5643853). Gender in groups was compared by Chi-square test and Fisher's exact test. IBM SPSS version 19.0 was used for statistical analysis. A *p* value of <0.05 was taken to indicate a statistically significant difference.

## 3. Results

### 3.1. Comparison of Urinary Neopterin Levels between Active and Latent* M. tuberculosis* Infection

**Figure 1:**
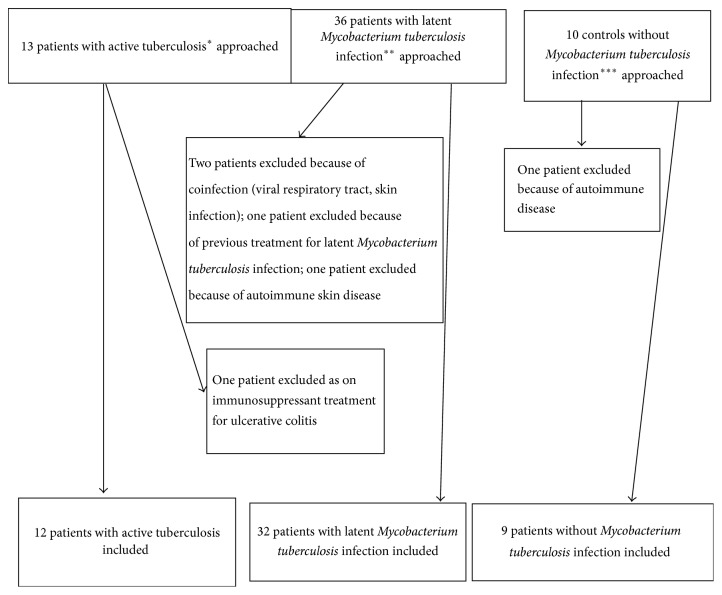
Flow chart of patient selection.  ^*∗*^Patients with positive tuberculin skin test or positive interferon-gamma release assay and clinical, radiological, or culture evidence of tuberculosis.  ^*∗∗*^Positive tuberculin skin test and positive interferon-gamma release assay or only a positive interferon-gamma release assay with no clinical, radiological, or culture evidence of tuberculosis.  ^*∗∗∗*^Healthy relatives testing negative by interferon-gamma release assay and/or tuberculin skin test during contact screening.

**Figure 2:**
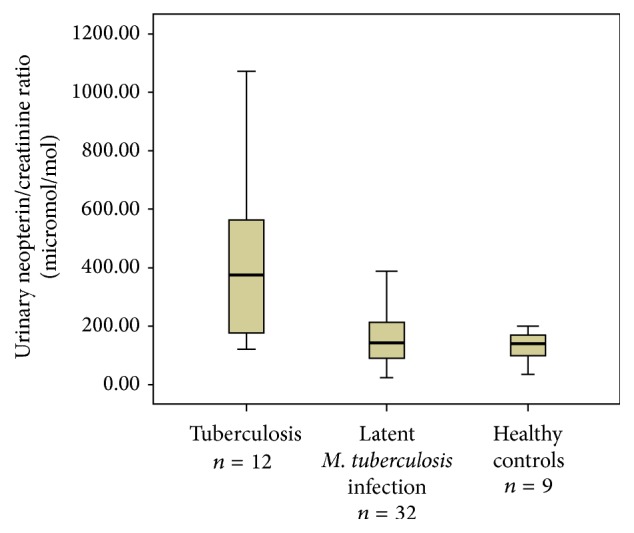
Boxplots with median (thick line within the box), interquartile range (box limits), and extremes (whiskers) of urinary neopterin/creatinine ratios in patients with active tuberculosis and latent* M. tuberculosis* infection and controls.

**Figure 3:**
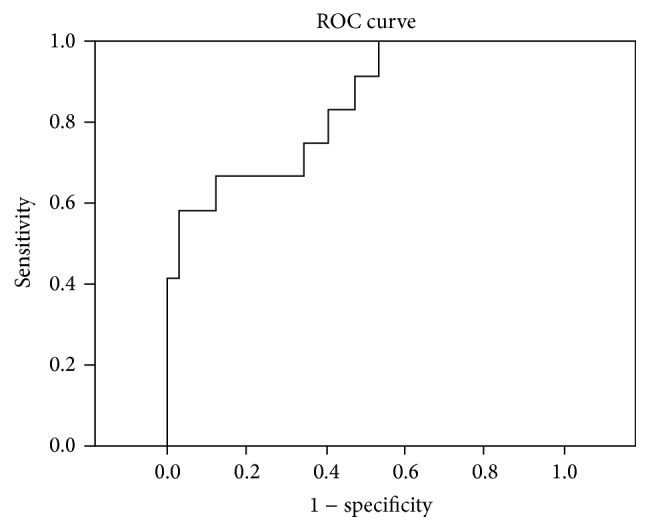
Receiver operating characteristics (ROC) curve depicting the relationship between sensitivity and specificity of urinary neopterin/creatinine ratios in detecting active tuberculosis.

We recruited 44 patients with* M. tuberculosis* infection out of which 12 had active tuberculosis. Amongst the patients with active tuberculosis, 9 patients had pulmonary and 3 patients had extrapulmonary tuberculosis (one each with tuberculous lymphadenitis, mandibular tuberculosis, and peritoneal tuberculosis). We also recruited nine healthy controls with negative interferon-gamma release assays on contact screening (for patient selection flow chart, see Figure 1). In 8/12 patients (66%), the tuberculosis was confirmed by culture. For patient characteristics and urinary neopterin/creatinine ratios, see Table 1. We included Supplementary Table 1 with the raw data for urinary neopterin levels, urinary creatinine levels, and urinary neopterin/creatinine ratios. None of the patients with active tuberculosis has been brought to our medical attention with a relapse at least two years after completion of the study and none of the patients with latent* M. tuberculosis* infection (LTBI) was diagnosed with active tuberculosis by our service at least two years after completion of treatment for latent infection. Age and gender were not significantly different between groups (*p* = 0.43 for age, female: active tuberculosis versus LTBI *p* = 0.77 and versus controls *p* = 1.0). Median urinary neopterin/creatinine ratio was significantly different between groups (*p* < 0.01) and significantly higher in patients with tuberculosis compared to latent* M. tuberculosis* infection and controls (*p* < 0.01) and not significantly different between patients with latent* M. tuberculosis* infection and controls (*p* = 0.66).  Figure 2 depicts boxplots of urinary neopterin/creatinine ratios in patients with tuberculosis and latent* M. tuberculosis* infection and healthy controls. To illustrate distribution of ratios in the groups compared, we included a supplementary figure with a dot plot (Supplementary Figure 1). To determine test accuracy in detection of active tuberculosis in all patients with* M. tuberculosis* infection, a receiver operating characteristics curve was generated (see Figure 3).

The receiver operating characteristics curve had an area under the curve of 0.84 (95% CI 0.70 to 0.97) which was significantly different (*p* < 0.01) from the null hypothesis that there was no discriminatory value of urinary neopterin/creatinine ratio in detecting active tuberculosis.

### 3.2. Change of Urinary Neopterin/Creatinine Ratio during Treatment

In a total of 22 patients with* M. tuberculosis* infection, urinary neopterin/creatinine ratios were repeated at a median of 3 months (interquartile range 3 to 6.8) after commencement of treatment which consisted in patients with active tuberculosis initially of a combination of isoniazid, rifampicin, pyrazinamide, and ethambutol for 2 months followed by 4 months of isoniazid and rifampicin and in patients with latent* M. tuberculosis* infection of isoniazid and rifampicin for 3 months. In six patients with active tuberculosis, urinary neopterin/creatinine ratio had a median (range) of 374.1 micromol/mol (174.3 to 1072.3) before treatment and a median of 200.2 (132 to 440.7) after commencement (*p* = 0.17). In the 16 patients with latent* M. tuberculosis* infection, median (range) urinary neopterin/creatinine ratio before treatment 211.95 micromol/mol (78.4 to 384.1) and during treatment 182 (67.2 to 420.8) (*p* = 0.87).

## 4. Discussion

The study presented is the first comparing urinary neopterin/creatinine ratios between patients with active and latent* M. tuberculosis* infection. It is to our knowledge also the first study investigating neopterin levels in patients with* M. tuberculosis* infection using an ELISA. Our study showed that urinary neopterin/creatinine ratio is a significant predictor of active tuberculosis in patients with* M. tuberculosis* infection. The range of urinary neopterin/creatinine ratios in patients with active tuberculosis overlapped ratios found in patients with latent* M. tuberculosis* infection and controls without* M. tuberculosis* infection. This may be due to patients with active tuberculosis showing suppression of the interferon-gamma release response by* M. tuberculosis* by increased activity of regulatory T-cells [10]. The patient with active tuberculosis with the lowest urinary neopterin/creatinine ratio had very limited unilateral axillary lymph node tuberculosis (culture positive) without systemic symptoms and without pulmonary involvement, which illustrates that limited extrapulmonary tuberculosis may be compatible with a normal urinary neopterin/creatinine ratio. The difference in urinary neopterin/creatinine ratio between active and latent* M. tuberculosis* infection found in this study is in line with previous studies showing a dependency of this ratio on the extent of tuberculous disease [7, 11]. Urinary neopterin/creatinine ratios are dependent on age, gender, and time of day of sampling. There was no difference in age and gender between groups and urine samples were taken during a morning clinic in all patients when levels are at their highest [12]. Previous studies on urinary neopterin/creatinine ratios in patients with* M. tuberculosis* infection used High Pressure Liquid Chromatography (HPLC). This methodological difference makes it difficult to compare the absolute levels found in previous studies with the levels detected in this study. Patients of our study with latent* M. tuberculosis* infection and controls without* M. tuberculosis* infection had similar median ratios to the mean ratios found by HPLC in healthy people described previously which ranged, dependent on age, from 101 to 226 micromol/mol [9]. Urinary neopterin/creatinine ratios determined by HPLC in 38 patients with active tuberculosis were reported previously [7]. The authors reported mean and standard deviation despite an apparently skewed distribution. An estimation of median levels from the scatter plot of this study points to a median of approximately 500 micromol/mol. This median is higher than the median of 374 micromol/mol found in our study.

A high urinary neopterin/creatinine ratio in the absence of other explanations in a symptomatic patient with normal chest X-ray should trigger further imaging like chest computer tomography (CT) and/or abdominal ultrasound to detect active tuberculosis. Future investigations need to establish whether, in the context of high infectious disease burden settings with endemic malaria, HIV, or helminth infections, which are all associated with elevation of neopterin levels [4], urinary neopterin/creatinine ratios remain a useful adjunct in ruling out active tuberculosis in a patient. As patients with active and latent* M. tuberculosis* infection may equally be affected by those background infections, a different baseline urinary neopterin/creatinine ratio in patients with latent* M. tuberculosis* infection may have to be established. A study conducted in asymptomatic outpatients in a hospital in Tanzania showed that serum neopterin levels were elevated above levels found in healthy people [13]. There is no evidence of an ethnic influence on neopterin levels as a study in low infectious disease burden urban populations in Lusaka, Zambia, demonstrated [14]. We did not find a significant difference in urinary neopterin/creatinine ratios before and during treatment in patients with active tuberculosis, which is likely due to the small sample size for this group. A sample size calculation showed that to detect a difference with 80% power at a significance level of 5% at least 16 patients were required.

In patients with latent* M. tuberculosis* infection, there was also no difference in neopterin levels before and during treatment. This may indicate that these patients had either eliminated the infection before treatment or a low grade infection is not associated with a significant elevation of urinary neopterin above the normal level for the individual patient and healthy controls. The low number of controls however was not able to exclude a statistically significant difference. A sample size calculation for design of an observational study proving equivalence of ratios showed that 52 people are required to exclude with a power of 80% a difference in means of more than 50 micromol/mol [15].

Assessment of accuracy of urinary neopterin/creatinine ratio in discriminating active from latent* M. tuberculosis* infection could be improved and precise cut-offs could be established by rigorous exclusion of occult pulmonary and extrapulmonary disease in patients labelled as having latent* M. tuberculosis* infection with detailed imaging like CT of the chest.

Future studies need to investigate urinary neopterin/creatinine ratios as a noninvasive tool to monitor treatment success early on and to serve as predictor of treatment failure and relapse particularly in patients infected with drug resistant* M. tuberculosis* or in patients infected with* M. tuberculosis* strains of unknown sensitivity. Clinicians and researchers hereby need to take into account that immune system activation involving stimulation of interferon-gamma release by T-effector cells as in autoimmune diseases like sarcoidosis or malignancies can increase urinary neopterin/creatinine ratios [16, 17]. Urinary neopterin/creatinine ratios could, in low income countries, if measured during treatment of pulmonary tuberculosis, help triage which patients, if the levels are not decreasing, require more expensive investigations like drug sensitivity testing and nucleic acid amplification testing.

## 5. Conclusions

Urinary neopterin/creatinine ratios are significantly higher in patients with active tuberculosis compared to patients with latent infection and may be a significant predictor of active tuberculosis in patients with* M. tuberculosis* infection.

## Supplementary Material

Appendix: Mean and standard deviation for urinary neopterin/creatinine ratios for patients with active and latent *M. tuberculosis* infection.
Table S1: Raw data for urinary neopterin levels, urinary creatinine levels, and urinary neopterin/creatinine ratios.
Figure S1: A dot plot to illustrate distribution of ratios in the groups compared.

## Figures and Tables

**Table 1:** 

	Patients with tuberculosis (*n* = 12)	Patients with latent *Mycobacterium tuberculosis* infection (*n* = 32)	Healthy controls (*n* = 9)
Age in years (median (range))	14.7 (5.8 to 37)	14.7 (4.5 to 49)	24 (9 to 40)

Gender (female)	7	19	6

Manifestations of disease	9 pulmonary tuberculosis, 3 extrapulmonary tuberculosis (1 each lymphadenitis, mandibular, and peritoneal)	No disease manifestations	No disease manifestations

Isolation of *Mycobacterium tuberculosis*	6/9 patients with pulmonary tuberculosis, and one each with tuberculous lymphadenitis, and mandibular tuberculosis	N/A	N/A

Urinary neopterin/creatinine ratio (micromole/mol, median (range))	374.1 (129.0 to 1072.3)	142.1 (28.0 to 384.1)	146.0 (40.3 to 200.0)
