# Supplementary material for: Determination of Urinary Neopterin/Creatinine Ratio to Distinguish Active Tuberculosis from Latent Mycobacterium tuberculosis Infection
Source: J Biomark. 2016 Jun 28;2016:5643853. doi: 10.1155/2016/5643853 (PMC4940561; doi:10.1155/2016/5643853)

Appendix

In tuberculosis mean neopterin/creatinine ratio= 429 micromol/mol standard deviation (SD)= 296, in latent M. tuberculosis infection: mean=162.1, SD=94.6, in controls: mean=134, SD=54.6.

In tuberculosis before treatment: mean (SD)= 503.9 (371.4) after treatment: mean= 233.9 (112.9),in latent M. tuberculosis infection before treatment: mean (SD) =213.3 (86.7) after treatment: mean = 217.2 (94.7).

Supplementary table 1. Raw data for urinary neopterin and creatinine levels and neopterin/creatinine ratios

	Urinary neopterin level (nmol/l)	Urinary creatinine level (mmol/l)	Urinary neopterin/ creatinine ratio (nmol/mmol)
Controls	1162	28.8	40.3
	384	5.5	69.8
	1061	8.1	130.9
	1162	11.5	101.0
	949	6.5	146.0
	1162	6.6	176.1
	2525	13.2	191.3
	474	3.l	153.1
	2020	10.1	200.0
Latent Mycobacterium tuberculosis infection	1121	9.1	123.0
	2222	25.1	88.5
	2020	14.4	140.0
	616	22.0	28.0
	1060	10.7	99.1
	313	1.8	173.9
	515	2.9	177.6
	1970	16.1	122.4
	1212	5.8	208.9
	990	8.8	112.5
	495	1.5	330.0
	1818	18.9	96.1
	1263	8.4	144.2
	1869	20.0	93.4
	1667	21.3	78.2
	657	11.8	55.7
	606	13.5	44.9
	707	18.8	37.6
	333	1.9	175.2
	929	8.7	106.8

	1616	22.0	73.4
	1030	4.1	251.2
	2525	10.6	238.2
	1081	3.6	300.3
	5252	16.3	322.2
	5151	28.0	184.0
	2727	7.1	384.1
	3030	9.6	315.6
	1818	8.2	221.7
	384	4.9	78.4
	3737	22.2	168.3
	1162	5.4	215.0
Active tuberculosis	1212	9.4	129.0
	5757	8.4	685.3
	6262	20.0	313.1
	1212	8.4	368.0
	2929	16.3	179.6
	889	5.1	174.3
	8686	8.1	1072.3
	3737	4.4	849.3
	3232	8.5	380.2
	3030	18.3	165.6
	6464	16.7	387.1
	2424	5.4	448.9

Supplementary Figure 1: Dot plot of urinary neopterin/creatinine ratios in patients with active tuberculosi[illegible] Mycobacterium tuberculosis infection and controls without Mycobacterium tuberculosis infection.

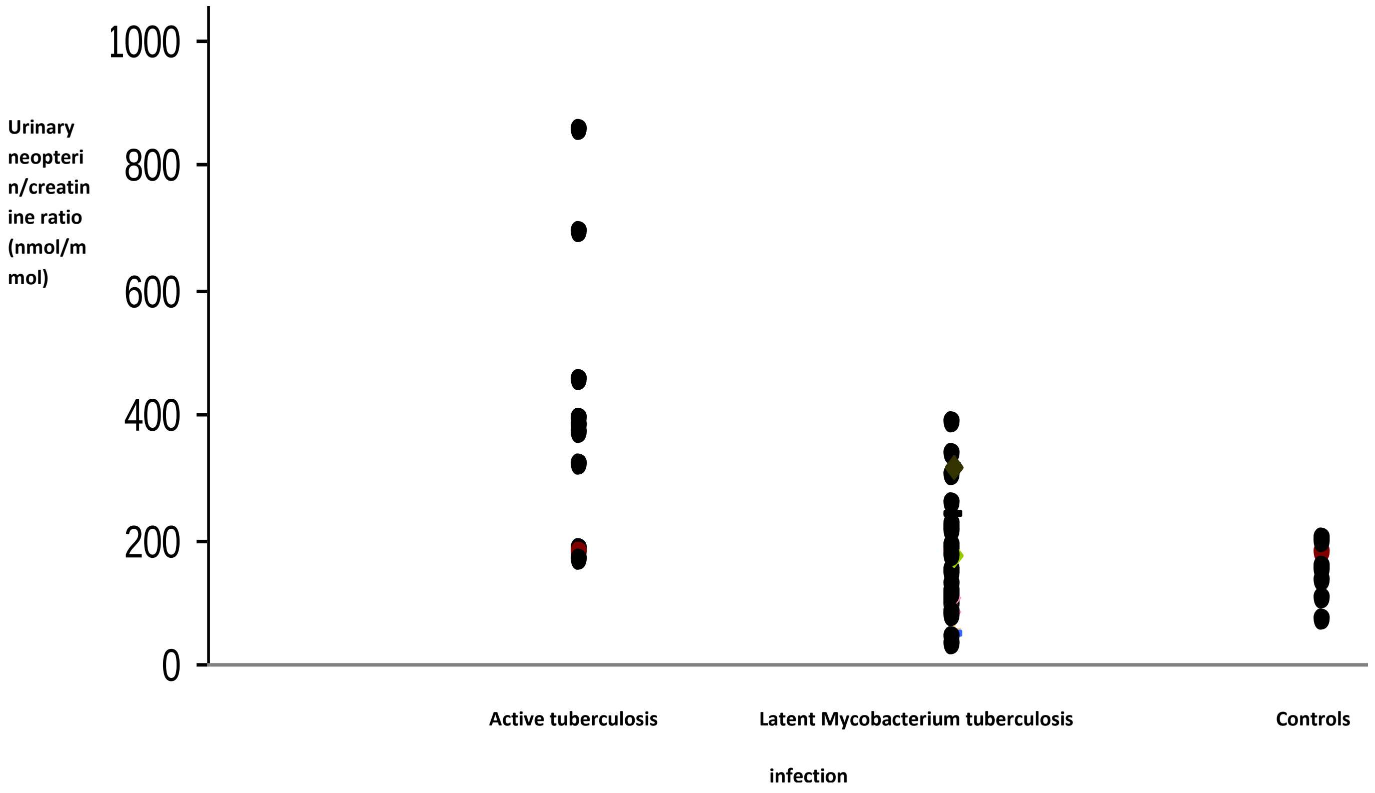

Supplement: Supplementary file 1 — Appendix: Mean and standard deviation for urinary neopterin/creatinine ratios for patients with active and latent M. tuberculosis infection. Table S1: Raw data for urinary neopterin levels, urinary creatinine levels, and urinary neopterin/creatinine ratios. Figure S1: A dot plot to illustrate distribution of ratios in the groups compared. [file 5643853.f1.pdf]
